# Roxithromycin treatment inhibits TGF-β1-induced activation of ERK and AKT and down-regulation of Caveolin-1 in rat airway smooth muscle cells

**DOI:** 10.1186/s12931-014-0096-z

**Published:** 2014-08-11

**Authors:** Yuanrong Dai, Fengqin Li, Liqin Wu, Ruili Wang, Ping Li, Sunshun Yan, Hui Xu, Mengling Xia, Chunxue Bai

**Affiliations:** Department of Pulmonary Medicine, the Second Affiliated Hospital of Wenzhou Medical University, Wenzhou, 325027 China; Department of Pulmonary Medicine, Research Institute of Respiratory Disease, Zhongshan Hospital, Fudan University, No. 180 Fenglin Road, Shanghai, 200032 China

**Keywords:** Airway smooth muscle cells, Roxithromycin, Caveolin-1, Transforming growth factor-β1, ERK1/2 pathway, Phosphatidylinositol 3-kinase/AKT pathway

## Abstract

**Background:**

Roxithromycin (RXM) has been widely used in asthma treatment; however, the mechanism has not been fully understood. The aim of our study was to investigate the underlying mechanism of RXM treatment in mediating the effect of transforming growth factor (TGF)-β1 on airway smooth muscle cells (ASMCs) proliferation and caveolinn-1 expression.

**Methods:**

Firstly, the rat ovalbumin (OVA) model was built according to the previous papers. Rat ASMCs were prepared and cultured, and then TGF-β1 production in ASMCs was measured by enzyme-linked immunosorbent assay (ELISA). Moreover, the proliferation of ASMCs was determined using cell counting kit (CCK-8) assay. Additionally, the expressions of caveolin-1, phosphorylated-ERK1/2 (p-ERK1/2) and phosphorylated–AKT (p-AKT) in ASMCs treated with or without PD98059 (an ERK1/2 inhibitor), wortannin (a PI3K inhibitor), β-cyclodextrin (β-CD) and RXM were measured by Western blot. Finally, data were evaluated using *t*–test or one-way ANOVA, and then a P value < 0.05 was set as a threshold.

**Results:**

Compared with normal control, TGF-β1 secretion was significantly increased in asthmatic ASMCs; meanwhile, TGF-β1 promoted ASMCs proliferation (P < 0.05). However, ASMCs proliferation was remarkably inhibited by RXM, β-CD, PD98059 and wortmannin (P < 0.05). Moreover, the expressions of p-ERK1/2 and p-AKT were increased and peaked at 20 min after TGF-β1 stimulation, and then suppressed by RXM. Further, caveolin-1 level was down-regulated by TGF-β1 and up-regulated by inhibitors and RXM.

**Conclusion:**

Our findings demonstrate that RXM treatment inhibits TGF-β1-induced activation of ERK and AKT and down-regulation of caveolin-1, which may be the potential mechanism of RXM protection from chronic inflammatory diseases, including bronchial asthma.

## Introduction

With inflammation being the principle underlying pathophysiological characteristic, asthma has been reported primarily as an inflammatory disorder, which would drive airway obstruction and remodeling [1]. Airway smooth muscle (ASM) has been discovered plays a central role in the pathogenesis of airway remodeling. Increased ASM mass has been shown to contribute to airway remodeling, which results in persistent airflow limitation [2]. Despite these studies have reported the potential pathogenesis of airway remodeling, the underlying mechanisms are not fully understood.

Previous studies have showed that transforming growth factor-β1 (TGF-β1) is involved in the pathophysiology of asthma [3]. Moreover, several researchers have discovered that elevated TGF-β1 expression is seen in the airway submucosa of asthmatic individuals compared with normal subjects; moreover, the TGF-β1 level is correlated with the severity of the disease [4]. In addition, TGF-β1 has reported increases the secretion of marix metalloproteinase [5] and production of extracellular matrix [6] in ASMCs, suggesting a new mechanism of airway remodeling. But the effect of TGF-β1 on the proliferation of ASMCs is still vague [7].

Roxithromycin (RXM), a fourteen-membered macrolide, enhances the phagocytic and bactericidal activities of neutrophils [8]. Clinical studies have shown its efficacy against chronic inflammatory diseases, including diffuse panbronchiolitis [9], chronic sinusitis [10], atopic dermatitis and bronchial asthma [11]. However, it is unclear if the beneficial effects are related to their anti-microbial or anti-proliferation properties. RXM may play an important role in TGFβ1-induced myofibroblast differentiation and collagen production [12]. To determine if RXM possesses anti-proliferation properties, the effect of RXM on ASMCs proliferation was examined in our present study; moreover, the potential mechanism of RXM treatment for asthma was analyzed.

Caveolae are flask-shaped plasma membrane characterized by high hydrophobicity. Many signal molecules, including caveolin-1, tyrosine kinase, Raf, MEK1/2, and transient receptor potential canonical (TRPC) accumulate in the caveolae [13]. Previous study has reported that caveolin-1 preventing asthmatic ASMCs proliferation is regulated by extracellular signal-regulated kinases 1 and 2 (ERK1/2) signal pathway, and then RXM reduces ASMCs proliferation by up-regulating caveolin-1 expression [14].

Proliferation is controlled by many cellular signaling pathways involving several serine/threoine kinase cascades, including the phosphatidylinositol 3-kinase (PI3K)/AKT and the mitogen-activated protein kinase (MAPK) pathways. AKT, also known as protein kinase B, is a major downstream target of PI3K activated in response to various stimuli, growth factors, and hormones. Another key mediator of growth is the ERK1/2. ERK1/2 belongs to the MAPK family and locates downstream of the cascade of Ras/Raf/MEK kinase. The nuclear translocation of ERK1/2 is a critical step for cell growth. In phosphorylated and activated forms, ERK1/2 transmits extracellular stimuli form the plasma membrane to the nucleus by phosphorylating and activating a variety of transcription factors [15]. One published study has reported that inhibition of ERK1/2 or PI3K/AKT signaling may attenuate ASM proliferation [16]. Based on the above research results, our study focused on investigating the mechanism of RXM treatment for chronic inflammatory diseases.

In the present study, the role of RAM in TGF-β1-induced activation of AKT and ERK1/2 and down-regulation of caveolin-1 was explored. Firstly, the effect of TGF-β1 and caveolin-1 on ASMCs proliferation was measured. Secondly, the time course of TGF-β1-induced activation of ERK1/2 and AKT was examined. Finally, the mechanism of RXM treatment in TGF-β1-induced ASMCs proliferation was analyzed. Our study investigated the potential role of RXM treatment in TGF-β1-induced asthma and analyzed the relationship between TGF-β1 and caveolin-1. This study set a novel insight in the mechanism of RXM treatment which may be helpful for chronic inflammatory diseases therapy.

## Materials and methods

### Materials and solutions

The recombinant human TGF-β1 was obtained from Peprotech (Rocky Hill, NJ, USA). The following reagents were purchased from Cell Signaling Technology Company (Boston, USA): rabbit anti-rat threonine/tyrosine diphosphorylated ERK1/2 and total ERK1/2 antibodies, MAPK inhibitor (PD98059), serine phosphorylated AKT, AKT and PI3K inhibitor (wortannin). Rabbit anti-rat caveolin-1 antibody was purchased from Abcam Company (Cambridge, UK). RXM and β-cyclodextrin (β-CD) were from Sigma Chemical Company (Santa Clara, CA, USA). TGF-β1 and RXM were taken pure water and dimethyl sulfoxide (DMSO) as their stock solutions, respectively. The final DMSO concentration of less than 0.2% had no significant effect on cells growth.

### Animals and experimental protocol

Male Sprague–Dawley (SD) rats (200–250 g, 6 weeks old) were purchased from SLAC Laboratory Animal Co., Ltd (Shanghai, China) and divided into control and asthma groups. The animals had no access to solid food but gained free access to water 12 hours before the experiments. The experimental protocol was approved by the Committee of Animal Care in Wenzhou Medical University. All animals were handled in accordance with the Guideline for the Care and Use of Laboratory Animals [17]. The ovalbumin (OVA) model was constructed as previously reported [18]: firstly, the rats were sensitized with intraperitoneal injection of 10% OVA mixed with 10% alumin hydroxide solution, and then from day 15, the rats were challenged for 30 min by an aerosol of 1% OVA in normal saline, twice a week for 8 weeks. In control group, OVA was replaced by saline during sensitization and challenge. After the last challenge, 10% chloral hydrate was used for all animals’ euthanasia.

### Preparation of ASMC_S_

ASMCs were isolated from male SD rats. Briefly, rat bronchi were isolated under sterile conditions. After the connective tissue and epithelia were removed carefully, the smooth muscle strips were cut into small pieces (<1 mm^3^) and digested in phosphate buffer saline (PBS), containing 0.2% type I collagenase (Sigma, USA), at 37°C in a 5% CO_2_ and 95% air atmosphere for 20 min. The cells were dispersed and centrifuged at 1000 × g for 5 min, and then the pellets were collected and resuspended in RPMI-1640, containing 10% FBS and 1% penicillin-streptomycin. Finally, the cultures were maintained in a humidified atmosphere with 5% CO_2_ at 37°C. Cells at passage 3–6 were used for experiments. In each group, total six rats were utilized and all experiments were repeated in cells from the six different rats. For all experiments, cells (5 × 10^5^) were plated into tissue culture flasks or Petri dishes and grown to 80% confluence. Furthermore, cells were serum-deprived for 24 hours in RPMI-1640 (containing 0.5% FBS) prior to the addition of TGF-β1.

### Stimuli and inhibitor

ASMCs were incubated in the low-serum medium with or without TGF-β1 (10 ng/ml) for 48 h. The inhibitors, including wortannin (a selective PI3K inhibitor) and PD98059 (a selective ERK1/2 inhibitor), and β-CD which could destroy caveolae (plasma membrane invaginations that regulate a variety of physiological functions) were used. Finally, 0.4 μM wortannin, 10 μM PD98059, and 10 mM β-CD were added to ASMCs 1 h prior to each treatment according to manufacturers’ instructions.

### Measurement of TGF-β1production

After centrifuged at 1500 × g for 5 min, ASMCs culture supernatants were collected and stored at −80°C. The concentrations of TGF-β1 in the supernatants were determined with a human TGF-β1 ELISA kit (Genzyme), according to the manufacturers’ instructions. Assays were run in triplicate and repeated twice.

### CCK-8 proliferation assay

Cell proliferation was determined using a cell counting kit (CCK-8, Beyotime Institute of Biotechnology, China), according to the manufacturers’ instructions. Briefly, cells were seeded at a density 5 × 10^3^ cells/well on 96-well plates and grown for the indicated time. After treatment, 10 μL of the CCK-8 solution was added into each well of the plate, followed by incubation for 2 h. Then, cell proliferation was determined by measuring absorbance at the wavelength of 450 nm in four samples of each group. Overall, total 9 groups, including control, asthma, TGF-β1, wortannin, TGF-β1 + wortannin, PD98059, TGF-β1 + PD98059, β-CD and TGF-β1 + β-CD, in this experiment.

### Morphological observation and Western blot analysis

Scanning electron microscope (SEM) was used to show caveolae structures and locations in ASMCs between control and OVA group. In addition, during Western bolt analysis, proteins were loaded onto each lane and separated by SDS-PAGE (15% gradient gels, Criterion Gel System; Bio-Rad, Hercules, CA), and then transferred onto polyvinylidene difluoride membranes (Millipore, Billerica, MA 01821, USA) for 30–60 min. After blocking with five percent nonfat milk in TBS containing 0.1% Tween 20 (TBST) for 1 h, membranes were incubated overnight at 4°C with primary antibodies (rabbit anti-caveolin-1, 1:1000; rabbit anti-p-akt, 1:1000; mouse anti-akt, 1:1000; rabbit anti-p-ERK1/2, 1:1000; mouse anti-ERK1/2, 1:1000; mouse anti-GAPDH, 1:5000). Following three washes with TBST, the membrane was incubated with horseradish peroxidase (HRP)-conjugated secondary antibodies (1:2000) for 1 h at room temperature and signals were developed by Gel Capture.

### The time course of ERK and AKT activation

To determine the effect of TGF-β1 stimulation on activation of ERK and AKT, the expression of these proteins on different time points (0, 5, 10, 15, and 60 min) were measured by Western blot. In detail, the expression of phosphorylated-ERK1/2 (p-ERK1/2) and phosphorylated-AKT (p-AKT) were assessed by Western blot and compared to the levels of ERK1/2 and AKT, respectively.

### The effect of RXM on p-ERK1/2, p-AKT and caveolin-1 levels

Cultured and serum-deprived asthmatic ASMCs were treated with TGF-β1 for 20 min for p-ERK1/2 and p-AKT measurement, with or without PD98059, wortmannin, β-CD and RXM (100 μg/ml). The expressions of p-ERK1/2 and p-AKT were measured. In addition, asthmatic ASMCs were treated with TGF-β1 for 48 h for caveolin-1 measurement, with or without PD98059, wortmannin, β-CD and RXM. Then the expression of caveolin-1 was detected.

### Statistical analysis

Bronchial samples from six rats were used to obtain ASMCs, with biochemistry and molecular biology protocols being repeated at least three times. Data were evaluated by *t*–test or one-way ANOVA for multiple comparisons. A P value < 0.05 was considered significant. All data were expressed as mean ± SD.

## Results

### TGF-β1 production in ASMCs and the effect of TGF-β1 on ASMCs proliferation

**Figure 1:**
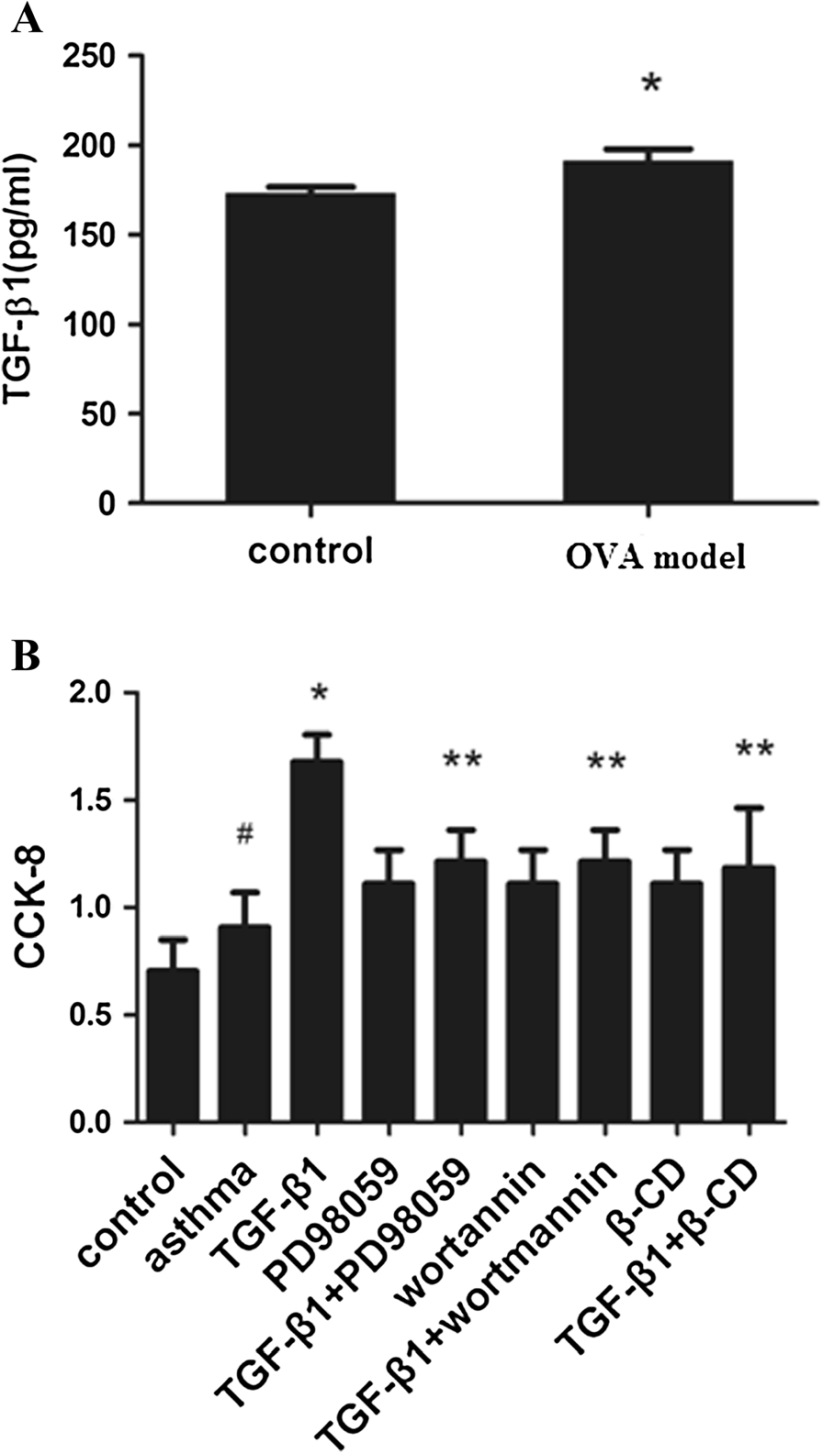
**TGF-β1 production in ASMCs and the effect of TGF-β1 on proliferation of ASMCs. (A)** Supernatants were collected after culture for 24 hours. Cytokine TGF-β1 was measured by ELISA. Data are expressed as mean ± SD for each group. (*P < 0.05 versus control). **(B)** Proliferation of ASMCs in each group was measure by CCK-8 assay. Data are expressed as mean ± SD for each group. TGF-β1 significantly promoted cell proliferation, which could be inhibited by PD98059, wortmannin and β-CD (*P < 0.05 versus control and asthma group, ^#^P < 0.05 versus control group, **P < 0.05 versus TGF-β1 group). No significant change between inhibitor group and TGF-β1 + inhibitor group was observed.

To determine the secretion of TGF-β1 in rat ASMCs, cells culture supernatants were collected and measured. Compared with control group, the secretion of TGF-β1 in OVA ASMCs was increased (P < 0.05, Figure 1A). Additionally, TGF-β1 treatment was discovered significantly promoted the proliferation of ASMCs (P < 0.05, Figure 1B). However, wortannin, PD98059 and β-CD alone or combination with TGF-β1 could inhibit the proliferation remarkably (P < 0.05). No significant difference was observed between inhibitor groups and TGF-β1 combining with inhibitor groups (Figure 1B).

### Caveolae and caveolin-1 in ASMCs

**Figure 2:**
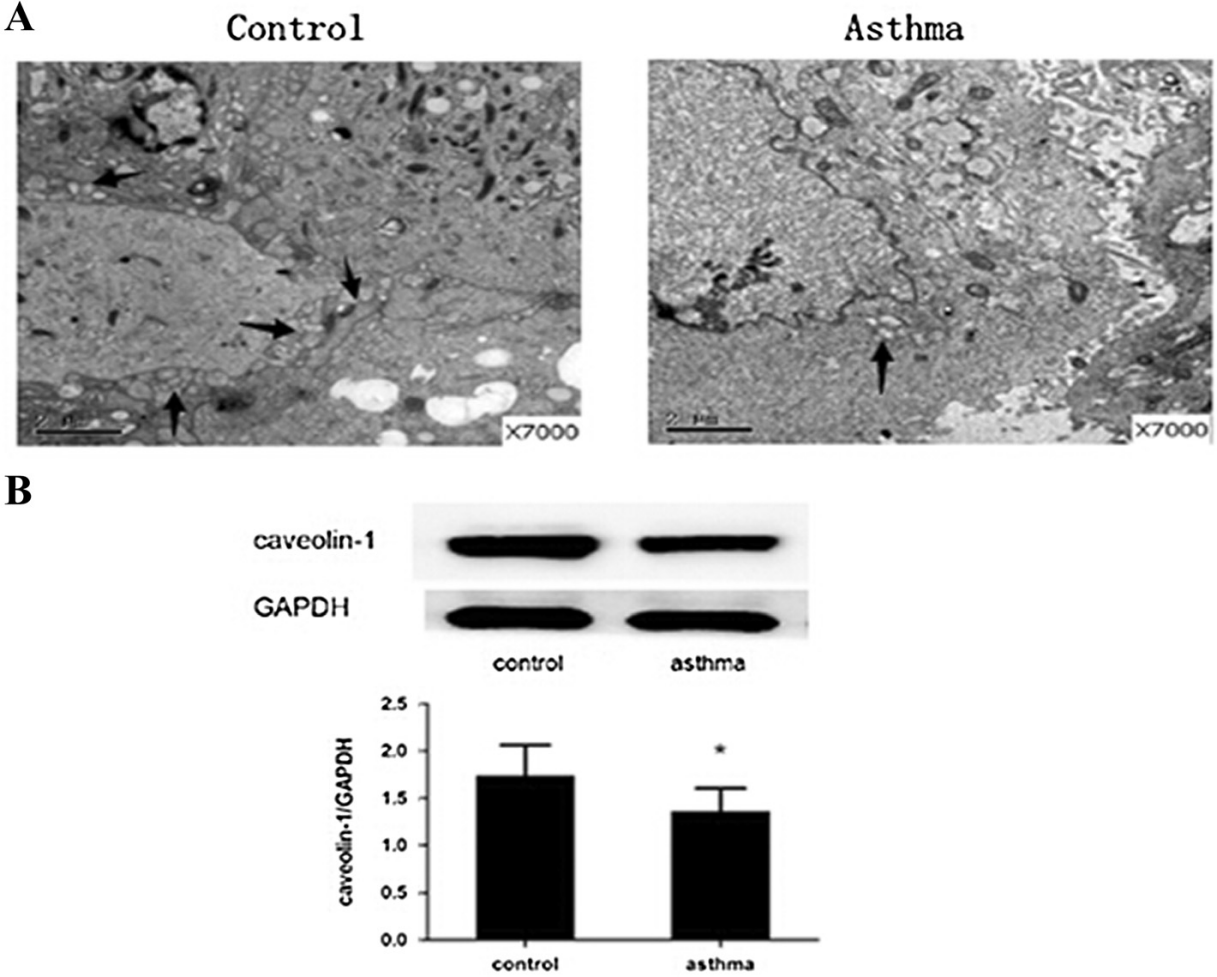
**The presence of caveolae and caveolin-1 in ASMCs. (A)** Normal caveolae structures were observed in the control group by scanning electron microscope (×7000). All the scale bars are 2 μm. However, there were fewer caveolae structures observed in the asthma group compared with control group. The locations of caveolae were indicated with arrows. **(B)** Western blot was used for analysis of caveolin-1 in separate group. GAPDH served as a loading control. The expression of caveolin-1 protein was significantly decreased in the asthma group. Data are expressed as mean ± SD for each group. (*P < 0.05 versus control).

By using SEM, we discovered that caveolae structures in ASMCs between control and asthmatic group showed significant differences. More caveolae were observed in control group than in asthmatic group (Figure 2A). Moreover, western blot was used to analyze caveolin-1 which mainly expressed in ASMCs. Compared with control group, the expression of caveolin-1 was significantly decreased in asthmatic group (P < 0.05, Figure 2B).

### The time course of ERK and AKT activation in ASMCs stimulated with TGF-β1

**Figure 3:**
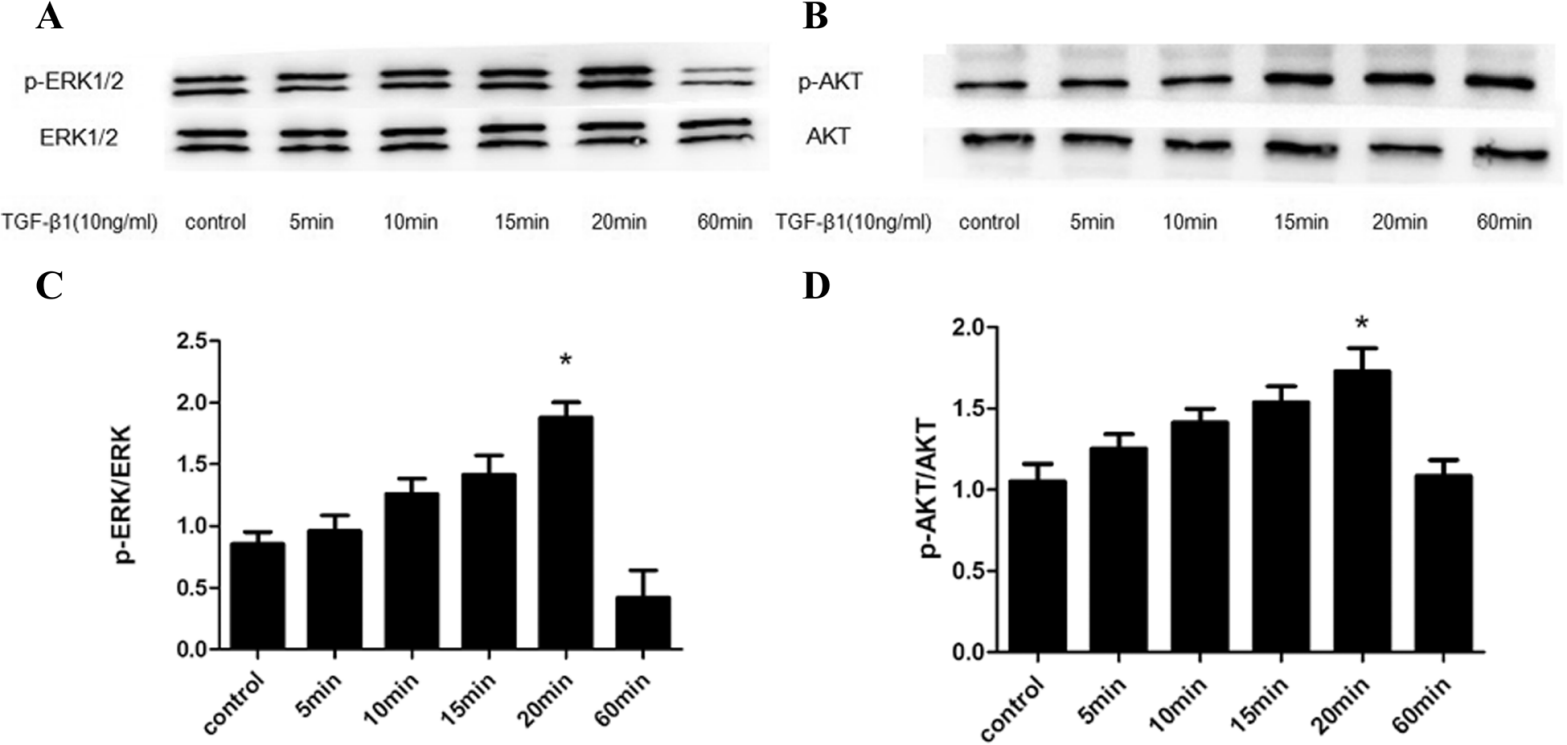
**The time course of ERK and AKT activation in ASMCs stimulated with TGF-β1. (A, C)** Asthmatic ASMCs were incubated with TGF-β1 (10 ng/ml) for 5, 10, 15, 20 and 60 min. The expression of p-ERK1/2 protein was assessed by western blot and analyzed by densitometry compared to ERK1/2 expression. The expression of p-ERK1/2 protein in ASMCs was increased and peaked at 20 min after TGF-β1 stimulation (*P < 0.05 versus control, 5, 10, 15, 60 min groups). **(B, D)** Asthmatic ASMCs were incubated with TGF-β1 (10 ng/ml) for 5, 10, 15, 20, and 60 min. The expression of p-AKT protein was assessed by western blot and analyzed by densitometry compared to AKT expression. The expression of p-AKT protein in ASMCs was increased and peaked at 20 min after TGF-β1 stimulation (*P < 0.05 versus control, 5, 10, 15, 60 min groups).

The effect of TGF-β1 on activation and expression of ERK and AKT was measured and our results indicated that ASMCs had a low level of p-ERK1/2 at the beginning of TGF-β1 stimulation. Over time, activation of ERK pathway reached to almost twofold of time zero at 20 min (P < 0.05), and then the level lowered and sustained at 60 min (Figure 3A, C). While, activation of AKT pathway reached its peak at 20 min and nearly maintained at the peak level at 60 min (Figure 3B, D).

### Mechanisms of TGF-β1-induced reduction in caveolin-1 and the effect of altered caveolin-1 expression on PI3K/AKT and ERK1/2 regulation

**Figure 4:**
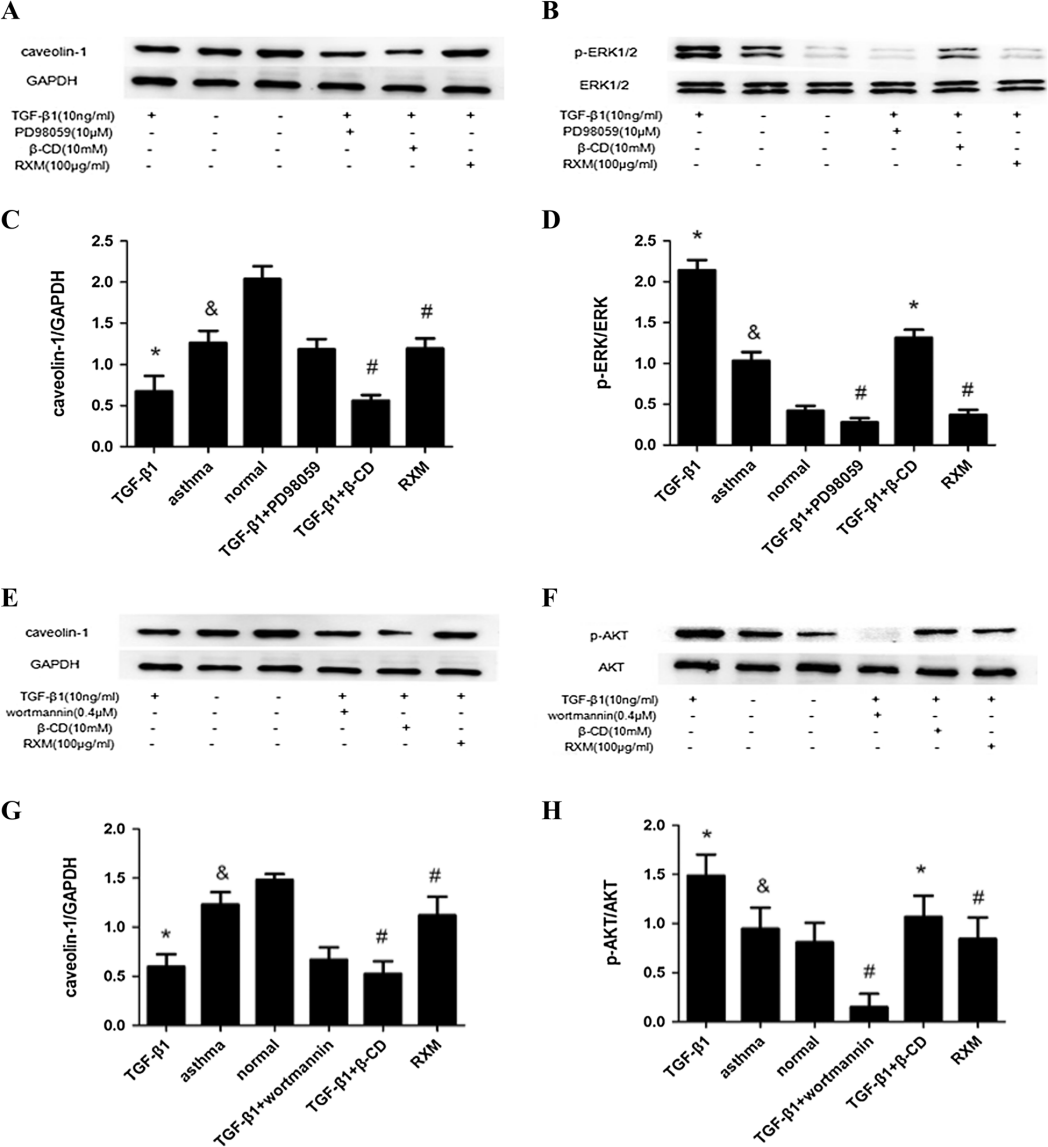
**Caveolin-1 suppressed expression of phosphorylated PI3K and ERK1/2 in ASMCs.** Cultured and serum-deprived ASMCs were treated with TGF-β1 for 20 min for p-ERK1/2 **(B, D)** and for p-AKT **(F, H)** measurement, with or without preincubation with PD98059, wortmannin, β-CD and RXM. The expressions of p-ERK1/2 and p-AKT were up-regulated by TGF-β1 and significantly down-regulated by PD98059, wortmannin, β-CD and RXM. **(A, C, E, G)** Asthmatic ASMCs were treated with TGF-β1 for 48 h for caveolin-1 measurement, with or without preincubation with PD98059, wortmannin, β-CD and RXM. The expression of caveolin-1 was down-regulated by TGF-β1and up-regulated by PD98059, wortmannin and RXM. Data are expressed as mean ± SD for each group (*P < 0.05 versus asthma and control group, & P < 0.05 versus control group, ^#^P < 0.05 versus TGF-β1 group).

Western blot analysis of ASMCs demonstrated a significantly reduced expression of caveolin-1 with TGF-β1 exposure (P < 0.05), and then caveolin-1 level was increased slightly after treated with either 10 μM PD98059 (ERK1/2 inhibitor) or 0.4 μM wortmannin (PI3K/AKT inhibitor) (Figure 4A, C, E, G). This result indicated that TGF-β1 inhibited caveolin-1 expression partly through PI3K and ERK1/2 pathways. In addition, the caveolae in cultured ASMCs were destroyed by β-CD with the expression of activated p-AKT and p-ERK1/2 significantly increased (P < 0.05), followed by decreased expression of caveolin-1 (P < 0.05) (Figure 4B, D, F, H). According to our results, decreased caveolin-1 expression was downstream of ERK and AKT activation. Thus, inhibition of ERK and AKT would inhibit TGF-β1-induced down-regulation of Caveolin-1.

### The effect of RXM on AKT and ERK1/2 activation and caveolin-1 expression

The effect of RXM on caveolin-1 expression in ASMCs was assessed by western blot. Compared with TGF-β1 group, RXM significantly increased the expression of caveolin-1 protein (P < 0.05) (Figure 4A, C, E, G). In addition, the expressions of p-AKT and p-ERK1/2 were significantly stimulated by TGF-β1 in ASMCs (P < 0.05), and remarkably down-regulated by RXM (P < 0.05) (Figure 4B, D, F, H). In a word, RXM treatment inhibited TGF-β1-induced activation of AKT and ERK1/2 and down-regulation of caveolin-1.

## Discussion

Our present study indicates that RXM treatment inhibits TGF-β1-induced activation of ERK and AKT and down-regulation of Caveolin-1 in ASMCs, which is an interesting novel finding about the potential mechanisms of RXM protection from chronic inflammatory diseases, including bronchial asthma seen in clinical studies. TGF-β1 is a central mediator in tissue remodeling processes, including fibrosis and ASM hyperplasia, as observed in asthma [19]. In our study, TGF-β1 secretion in ASMCs from OVA rats was increased, which promoted ASMCs themselves proliferation significantly. Different mechanisms have been suggested to explain the role of TGF-β1 in ASMCs proliferation. While, our present study found TGF-β1 reduced caveolin-1 expression on plasma membrane and promoted its partial translocation to the cytoplasm. Moreover, the inactivation of caveolin-1 would unlock a negative regulation process which allows TGF-β1 to promote ASMCs proliferation. This may be the new mechanism about TGF-β1 affecting ASMCs biological processes.

Caveolae are flask-shaped plasma membrane invaginations rich in cholesterol and sphingolipids. They express any of three caveolin proteins (caveolin-1, caveolin-2, and/or caveolin-3) and contain agonist receptors, ion channels, and other membrane proteins [20]. Caveolin-1 is a main component of caveolae. Studies using gene knockout technology indicate that caveolin-1 plays a key role in the formation and mobility of the caveolae [21]. In our present study, caveolae and caveolin-1 were few in asthmatic ASMCs, while they were abundant in normal cells. Absence of caveolin-1, an essential protein for caveolae formation on the plasma membrane, abrogated TGF-β1 mediated phosphorylation of AKT, which culminated in decreased ASMCs proliferation. In addition, recent studies have proposed that TGF-β mediates AKT activation is facilitated via EGFR (epidermal growth factor receptor) ligand secretion, EGFR activation and subsequent c-Src phosphorylation [22,23]. However, caveolin-1 was shown to counteract EGFR signaling [24,25]. Additionally, PI3K/AKT signaling has recently been shown to require lipid raft compartments (where caveolin-1 is usually located) to enable activation [26]. Our further research activities will shed insight on how TGF-β induces AKT in a caveolin-1 dependent manner.

Other papers have shown that caveolae structures are required for ERK1/2 activation and ERK1/2 activation is decreased after the caveolae structure is destroyed by removing cholesterol from the plasma membrane using β-CD, which in turn inhibits cell proliferation [27,28]. Furthermore, studies using cholesterol to increase ERK1/2 activation resulted in an increase in cell proliferation. These findings suggest that caveolae collect ERK1/2 and other signal molecules resulting in the activation of phosphorylated cascade of ERK1/2, indicating that caveolae might be pivotal for the generation of signaling mechanisms that trigger cell proliferation. In our study, β-CD was discovered inhibited TGF-β1-induced ASMCs proliferation and decreased p-ERK1/2 and p-AKT expression, suggesting that an intact caveolae were required for TGF-β1-induced ASMCs proliferation, ERK1/2 and AKT activation.

In ASMCs, following TGF-β1 stimulation, both PI3K/AKT and ERK1/2 pathways were activated independently and play roles in cell survival. Further researches suggest an enhanced Raf/MEK/ERK effect upon PI3K/AKT activation [29,30]. The stimulation of both pathways by many common ligands raises the possibility that cross-talking between the PI3K/AKT and ERK1/2 pathways could play a major role in regulating cell proliferation under particular conditions. Cross-talking between PI3K/AKT and Ras/Raf/MEK/ERK1/2 occurs at different levels and exerts cooperative or antagonistic effects depending on external stimuli and cellular background. Cooperative effects between these two signaling cascades have also been demonstrated in the regulation of platelet-derived growth factor-induced proliferation. In our study, exposure of ASMCs to TGF-β1 resulted in PI3K/AKT and ERK1/2 pathways activation with similar time course. To date, the molecular mechanisms and functional cellular consequences of this cross-talking remain poorly investigated.

Interestingly, our results also suggest that RXM suppresses ASMCs proliferation stimulated with TGF-β1 and caveolin-1 down-expression induced by TGF-β1. The present study reveals that RXM inhibits activation of PI3K/AKT and ERK1/2 pathways. Sohshi *et al.* have reported that RXM has the ability to inhibit phosphorylation of AKT and ERK of EL-4 cells [31]. Zeng *et al.* discover that RXM suppresses asthmatic ASMCs through up-regulating caveolin-1 expression and inhibiting monocyte chemotactic protein-1 expression [14]. In addition, RXM has the ability to inhibit TNF-α-mediated matrix metalloproteinase (MMP)-1 induction through ERK1/2 down-regulation, and then treats MMP-1 induced chronic inflammation diseases [32]. On the other hand, it has shown that RXM has an effect on the cyclin-dependent kinases (CDKs) activities and the expression of cell cycle regulatory proteins. RXM could clearly suppress both CDK4 and CDK2 activities without affecting their protein levels. The reduction of CDK4 and CDK2 activities is likely due to the decreased expression of cyclin D1 and cyclin A, and inhibition of p27 down-regulation. Moreover, RXM inhibits human coronary artery smooth muscle cells (CASMCs) proliferation by modulating cell cycle regulatory proteins and suppressing NF-kappaB signaling pathway [33]. Overall, our findings are consistent with these previous studies and reveal a novel mechanism of RXM treatment for chronic inflammation disease, including asthma.

## Conclusion

In conclusion, the results of the present study demonstrate that RXM treatment inhibits TGF-β1-induced ASMCs proliferation which may suppress activation of PI3K/AKT and ERK1/2 activation and caveolin-1 down-expression. This anti-proliferative effect of RXM would propose a novel beneficial mechanism in clinical trials using antibiotics. In next study, we plan to explore the role of RXM in TGF-β1 or caveolin-1 expression and ASMC remodeling in vivo. Moreover, the expression of TGF-β1 and caveolin-1 will be examined by either immunostaining or hybridization in our next experiment. Additionally, caveolin-1 siRNA or TGF-β1 receptor inhibition will be used to address the underlying signaling mechanisms in our plan.

## Abbreviations

ASMCs: Airway smooth muscle cells
TGF- β1: Transforming growth factor
RXM: Roxithromycin
DMSO: Dimethyl sulfoxide
β-CD: β-cyclodextrin
RPMI-1640: Roswell park memorial institute −1640
SDS: Sodium dodecylsulfate
PAGE: Polyacrylamide gel electrophoresis
